# [μ-1,2,3,4-Tetra­kis(pyridin-4-yl)butane-κ^2^ *N* ^1^:*N* ^4^]bis­[trimeth­yl(thio­cyanato-κ*N*)tin(IV)]

**DOI:** 10.1107/S160053681204737X

**Published:** 2012-11-28

**Authors:** Ezzatollah Najafi, Mostafa M. Amini, Seik Weng Ng

**Affiliations:** a Department of Chemistry, General Campus, Shahid Beheshti University, Tehran 1983963113, Iran; b Department of Chemistry, University of Malaya, 50603 Kuala Lumpur, Malaysia; c Chemistry Department, Faculty of Science, King Abdulaziz University, PO Box 80203 Jeddah, Saudi Arabia

## Abstract

In the title compound, [Sn_2_(CH_3_)_6_(NCS)_2_(C_24_H_22_N_4_)], the 1,2,3,4-tetra­kis­(pyridin-4-yl)butane ligand uses the pyridine N atoms at the ends of the butyl chain to coordinate to two trimethylthiocyanatotin(IV) units, forming a dinuclear structure. The Sn^IV^ atom in the mol­ecule shows a distorted *trans*-trigonal–bipyramidal coordination with the methyl groups in equatorial positions. The mol­ecule lies on a center of inversion, with the mid-point of the butyl chain coinciding with this symmetry element. In the crystal, weak C—H⋯π inter­actions occur between pyridine rings of adjacent mol­ecules.

## Related literature

For trimethyl­tin(IV) thio­cyanate, see: Forder & Sheldrick (1970 ▶).
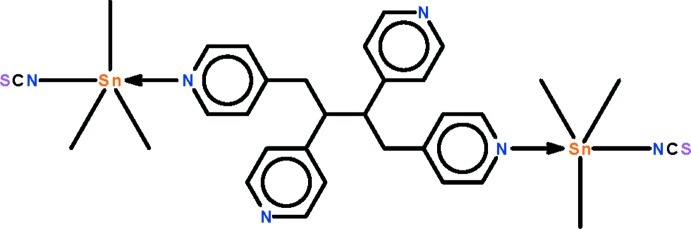


## Experimental

### 

#### Crystal data

[Sn_2_(CH_3_)_6_(NCS)_2_(C_24_H_22_N_4_)]
*M* *_r_* = 810.20
Triclinic, 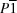

*a* = 9.2959 (8) Å
*b* = 9.7210 (7) Å
*c* = 10.2448 (9) Å
α = 90.388 (7)°
β = 94.381 (7)°
γ = 103.646 (7)°
*V* = 896.72 (13) Å^3^
*Z* = 1
Mo *K*α radiation
μ = 1.54 mm^−1^
*T* = 295 K
0.25 × 0.25 × 0.05 mm

#### Data collection

Agilent SuperNova Dual diffractometer with an Atlas detector
Absorption correction: multi-scan (*CrysAlis PRO*; Agilent, 2012 ▶) *T*_min_ = 0.700, *T*_max_ = 0.927
8527 measured reflections
4153 independent reflections
3645 reflections with *I* > 2σ(*I*)
*R*_int_ = 0.030

#### Refinement

*R*[*F* ^2^ > 2σ(*F* ^2^)] = 0.031
*wR*(*F* ^2^) = 0.076
*S* = 1.05
4153 reflections
190 parameters
H-atom parameters constrained
Δρ_max_ = 0.50 e Å^−3^
Δρ_min_ = −0.52 e Å^−3^

### 

Data collection: *CrysAlis PRO* (Agilent, 2012 ▶); cell refinement: *CrysAlis PRO*; data reduction: *CrysAlis PRO*; program(s) used to solve structure: *SHELXS97* (Sheldrick, 2008 ▶); program(s) used to refine structure: *SHELXL97* (Sheldrick, 2008 ▶); molecular graphics: *X-SEED* (Barbour, 2001 ▶); software used to prepare material for publication: *publCIF* (Westrip, 2010 ▶).

## Supplementary Material

Crystal structure: contains datablock(s) global, I. DOI: 10.1107/S160053681204737X/xu5652sup1.cif

Structure factors: contains datablock(s) I. DOI: 10.1107/S160053681204737X/xu5652Isup2.hkl

Additional supplementary materials:  crystallographic information; 3D view; checkCIF report

## Figures and Tables

**Table 1:** Hydrogen-bond geometry (Å, °) *Cg* is the centroid of the N2-pyridine ring.

*D*—H⋯*A*	*D*—H	H⋯*A*	*D*⋯*A*	*D*—H⋯*A*
C14—H14⋯*Cg* ^i^	0.93	2.79	3.631 (4)	151

Symmetry code: (i) 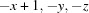.

**Fig. 1.**
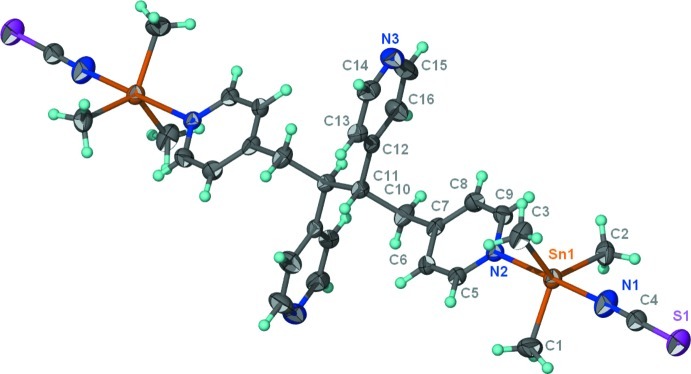
Thermal ellipsoid plot (Barbour, 2001) of [(CH3)3Sn(NCS)]2(C24H22N4) at the 50% probability level; hydrogen atoms are drawn as spheres of arbitrary radius.

## supplementary crystallographic information

### Comment

Unlike trimethyltin chloride, the pseudohalide, trimethyltin thiocyanate, furnishes only few coordination compounds with aromatic amines. Trimethyltin thiocyanate itself exists as a zigzag chain in which the thiocyanate unit bridges adjacent trimethyltin cations (Forder & Sheldrick, 1970). The title adduct (Scheme I, Fig. 1) is the first crystal structure report of such an adduct. The tetrapyridyl-substitutent butane ligand, C_24_H_22_N_4_, uses the pyridine N-atoms at the either ends of the butyl chain to coordinate to a trimethylthiocyanatotin unit. The dinuclear molecule lies on a center-of-inversion, with the mid-point of the butyl chain coinciding with this symmetry element.

The Sn atom is displaced out of the trigonal plane, in the direction of the thiocyanate ion, by 0.036 (2) Å.

### Experimental

Trimethyltin thiocyanate (0.19 g, 1 mmol) and 4-[1,3,4-tris(pyridin-4-yl)butan-2-yl]pyridine (0.73 g, 2 mmol) were loaded into a convection tube; the tube was filled with ethyl alcohol andkept at 333 K. Colorless crystals were collected from the side arm after several days.

### Refinement

Carbon-bound H atoms were placed in calculated positions [C–H 0.93 to 0.96 Å, *U*_iso_(H) 1.2 to 1.5*U*_eq_(C)] and were included in the refinement in the riding model approximation.

### Crystal data

**Table:**

[Sn_2_(CH_3_)_6_(NCS)_2_(C_24_H_22_N_4_)]	*Z* = 1
*M**_r_* = 810.20	*F*(000) = 406
Triclinic, *P*1	*D*_x_ = 1.500 Mg m^−^^3^
Hall symbol: -P 1	Mo *K*α radiation, λ = 0.71073 Å
*a* = 9.2959 (8) Å	Cell parameters from 3676 reflections
*b* = 9.7210 (7) Å	θ = 2.9–27.5°
*c* = 10.2448 (9) Å	µ = 1.54 mm^−^^1^
α = 90.388 (7)°	*T* = 295 K
β = 94.381 (7)°	Prism, colorless
γ = 103.646 (7)°	0.25 × 0.25 × 0.05 mm
*V* = 896.72 (13) Å^3^	

### Data collection

**Table:**

Agilent SuperNova Dual diffractometer with an Atlas detector	4153 independent reflections
Radiation source: SuperNova (Mo) X-ray Source	3645 reflections with *I* > 2σ(*I*)
Mirror monochromator	*R*_int_ = 0.030
Detector resolution: 10.4041 pixels mm^-1^	θ_max_ = 27.6°, θ_min_ = 2.9°
ω scan	*h* = −10→12
Absorption correction: multi-scan (*CrysAlis PRO*; Agilent, 2012)	*k* = −12→12
*T*_min_ = 0.700, *T*_max_ = 0.927	*l* = −12→13
8527 measured reflections	

### Refinement

**Table:**

Refinement on *F*^2^	Primary atom site location: structure-invariant direct methods
Least-squares matrix: full	Secondary atom site location: difference Fourier map
*R*[*F*^2^ > 2σ(*F*^2^)] = 0.031	Hydrogen site location: inferred from neighbouring sites
*wR*(*F*^2^) = 0.076	H-atom parameters constrained
*S* = 1.05	*w* = 1/[σ^2^(*F*_o_^2^) + (0.0324*P*)^2^ + 0.1997*P*] where *P* = (*F*_o_^2^ + 2*F*_c_^2^)/3
4153 reflections	(Δ/σ)_max_ = 0.001
190 parameters	Δρ_max_ = 0.50 e Å^−^^3^
0 restraints	Δρ_min_ = −0.52 e Å^−^^3^

### Fractional atomic coordinates and isotropic or equivalent isotropic displacement parameters (Å^2^)

**Table:**

	*x*	*y*	*z*	*U*_iso_*/*U*_eq_	
Sn1	0.76200 (2)	0.553034 (18)	0.347974 (18)	0.03890 (8)	
S1	1.20140 (12)	0.90346 (10)	0.55879 (11)	0.0742 (3)	
N1	0.9749 (3)	0.7032 (3)	0.4286 (3)	0.0656 (9)	
N2	0.5239 (3)	0.3899 (2)	0.2637 (2)	0.0347 (5)	
N3	0.3574 (3)	−0.2627 (3)	0.0683 (3)	0.0566 (7)	
C1	0.6775 (5)	0.7271 (3)	0.2837 (4)	0.0666 (11)	
H1A	0.7503	0.8136	0.3056	0.100*	
H1B	0.5888	0.7273	0.3259	0.100*	
H1C	0.6549	0.7193	0.1906	0.100*	
C2	0.7196 (4)	0.4771 (3)	0.5374 (3)	0.0531 (8)	
H2A	0.7987	0.5248	0.5993	0.080*	
H2B	0.7133	0.3771	0.5381	0.080*	
H2C	0.6275	0.4948	0.5608	0.080*	
C3	0.8759 (4)	0.4507 (4)	0.2210 (3)	0.0579 (9)	
H3A	0.9803	0.4938	0.2321	0.087*	
H3B	0.8397	0.4597	0.1319	0.087*	
H3C	0.8592	0.3522	0.2414	0.087*	
C4	1.0677 (4)	0.7876 (3)	0.4841 (3)	0.0458 (7)	
C5	0.4359 (3)	0.4259 (3)	0.1683 (3)	0.0382 (6)	
H5	0.4698	0.5106	0.1264	0.046*	
C6	0.2962 (3)	0.3436 (3)	0.1280 (3)	0.0403 (6)	
H6	0.2388	0.3734	0.0606	0.048*	
C7	0.2420 (3)	0.2165 (3)	0.1887 (3)	0.0351 (6)	
C8	0.3340 (3)	0.1789 (3)	0.2859 (3)	0.0437 (7)	
H8	0.3030	0.0944	0.3290	0.052*	
C9	0.4726 (3)	0.2655 (3)	0.3204 (3)	0.0439 (7)	
H9	0.5332	0.2365	0.3857	0.053*	
C10	0.0880 (3)	0.1277 (3)	0.1497 (3)	0.0427 (7)	
H10A	0.0202	0.1894	0.1367	0.051*	
H10B	0.0551	0.0649	0.2203	0.051*	
C11	0.0817 (3)	0.0381 (2)	0.0226 (3)	0.0315 (5)	
H11	0.1201	0.1030	−0.0463	0.038*	
C12	0.1783 (3)	−0.0664 (3)	0.0395 (2)	0.0314 (5)	
C13	0.2892 (3)	−0.0683 (3)	−0.0411 (3)	0.0373 (6)	
H13	0.3068	−0.0037	−0.1079	0.045*	
C14	0.3750 (4)	−0.1665 (3)	−0.0230 (3)	0.0483 (7)	
H14	0.4498	−0.1644	−0.0789	0.058*	
C15	0.2501 (5)	−0.2597 (4)	0.1456 (4)	0.0619 (10)	
H15	0.2353	−0.3257	0.2115	0.074*	
C16	0.1589 (4)	−0.1668 (3)	0.1362 (3)	0.0510 (8)	
H16	0.0854	−0.1712	0.1939	0.061*	

### Atomic displacement parameters (Å^2^)

**Table:**

	*U*^11^	*U*^22^	*U*^33^	*U*^12^	*U*^13^	*U*^23^
Sn1	0.03404 (13)	0.03835 (12)	0.04120 (12)	0.00409 (8)	−0.00196 (8)	−0.00293 (8)
S1	0.0695 (7)	0.0535 (5)	0.0823 (7)	−0.0110 (5)	−0.0211 (5)	−0.0118 (4)
N1	0.0449 (18)	0.0651 (18)	0.076 (2)	−0.0031 (15)	−0.0090 (15)	−0.0174 (15)
N2	0.0307 (12)	0.0330 (11)	0.0389 (12)	0.0063 (9)	−0.0018 (9)	−0.0036 (9)
N3	0.0480 (18)	0.0529 (16)	0.072 (2)	0.0224 (13)	−0.0095 (15)	−0.0021 (14)
C1	0.071 (3)	0.0364 (16)	0.086 (3)	0.0074 (16)	−0.018 (2)	0.0022 (15)
C2	0.056 (2)	0.066 (2)	0.0380 (16)	0.0187 (16)	−0.0005 (14)	−0.0060 (14)
C3	0.0422 (19)	0.070 (2)	0.058 (2)	0.0038 (16)	0.0117 (15)	−0.0171 (16)
C4	0.0440 (18)	0.0423 (15)	0.0475 (17)	0.0043 (14)	−0.0004 (14)	−0.0002 (12)
C5	0.0411 (16)	0.0313 (13)	0.0384 (14)	0.0028 (11)	−0.0019 (12)	0.0001 (10)
C6	0.0413 (17)	0.0400 (14)	0.0388 (15)	0.0126 (12)	−0.0102 (12)	−0.0044 (11)
C7	0.0282 (14)	0.0332 (13)	0.0416 (14)	0.0035 (11)	0.0010 (11)	−0.0113 (10)
C8	0.0444 (18)	0.0332 (13)	0.0480 (16)	−0.0003 (12)	−0.0025 (13)	0.0029 (11)
C9	0.0397 (17)	0.0365 (14)	0.0511 (17)	0.0053 (12)	−0.0116 (13)	0.0025 (12)
C10	0.0279 (15)	0.0472 (15)	0.0509 (17)	0.0054 (12)	0.0017 (12)	−0.0149 (12)
C11	0.0221 (13)	0.0306 (12)	0.0394 (13)	0.0020 (10)	0.0011 (10)	−0.0032 (10)
C12	0.0245 (13)	0.0342 (12)	0.0333 (13)	0.0048 (10)	−0.0038 (10)	−0.0044 (10)
C13	0.0323 (15)	0.0351 (13)	0.0438 (15)	0.0065 (11)	0.0041 (12)	0.0003 (11)
C14	0.0366 (17)	0.0446 (16)	0.065 (2)	0.0120 (13)	0.0039 (14)	−0.0089 (14)
C15	0.068 (3)	0.0549 (19)	0.064 (2)	0.0211 (18)	−0.0032 (19)	0.0193 (16)
C16	0.050 (2)	0.0559 (18)	0.0497 (18)	0.0151 (15)	0.0096 (15)	0.0136 (14)

### Geometric parameters (Å, º)

**Table:**

Sn1—C1	2.116 (3)	C6—C7	1.390 (4)
Sn1—C3	2.119 (3)	C6—H6	0.9300
Sn1—C2	2.113 (3)	C7—C8	1.371 (4)
Sn1—N1	2.258 (3)	C7—C10	1.509 (4)
Sn1—N2	2.489 (2)	C8—C9	1.381 (4)
S1—C4	1.606 (3)	C8—H8	0.9300
N1—C4	1.152 (4)	C9—H9	0.9300
N2—C5	1.328 (3)	C10—C11	1.551 (4)
N2—C9	1.344 (3)	C10—H10A	0.9700
N3—C14	1.319 (4)	C10—H10B	0.9700
N3—C15	1.326 (5)	C11—C12	1.509 (4)
C1—H1A	0.9600	C11—C11^i^	1.557 (5)
C1—H1B	0.9600	C11—H11	0.9800
C1—H1C	0.9600	C12—C13	1.373 (4)
C2—H2A	0.9600	C12—C16	1.388 (4)
C2—H2B	0.9600	C13—C14	1.385 (4)
C2—H2C	0.9600	C13—H13	0.9300
C3—H3A	0.9600	C14—H14	0.9300
C3—H3B	0.9600	C15—C16	1.376 (5)
C3—H3C	0.9600	C15—H15	0.9300
C5—C6	1.386 (4)	C16—H16	0.9300
C5—H5	0.9300		
			
C1—Sn1—C3	120.57 (17)	C5—C6—H6	120.1
C1—Sn1—C2	118.37 (16)	C7—C6—H6	120.1
C3—Sn1—C2	120.97 (15)	C8—C7—C6	116.6 (2)
C1—Sn1—N1	90.01 (13)	C8—C7—C10	122.7 (2)
C3—Sn1—N1	92.20 (13)	C6—C7—C10	120.7 (3)
C2—Sn1—N1	90.71 (12)	C7—C8—C9	120.5 (2)
C1—Sn1—N2	89.41 (11)	C7—C8—H8	119.8
C3—Sn1—N2	89.29 (10)	C9—C8—H8	119.8
C2—Sn1—N2	88.33 (10)	N2—C9—C8	123.0 (3)
N1—Sn1—N2	178.49 (10)	N2—C9—H9	118.5
C4—N1—Sn1	168.1 (3)	C8—C9—H9	118.5
C5—N2—C9	116.8 (2)	C7—C10—C11	112.5 (2)
C5—N2—Sn1	121.93 (16)	C7—C10—H10A	109.1
C9—N2—Sn1	121.08 (18)	C11—C10—H10A	109.1
C14—N3—C15	115.2 (3)	C7—C10—H10B	109.1
Sn1—C1—H1A	109.5	C11—C10—H10B	109.1
Sn1—C1—H1B	109.5	H10A—C10—H10B	107.8
H1A—C1—H1B	109.5	C12—C11—C10	111.5 (2)
Sn1—C1—H1C	109.5	C12—C11—C11^i^	111.1 (2)
H1A—C1—H1C	109.5	C10—C11—C11^i^	110.7 (3)
H1B—C1—H1C	109.5	C12—C11—H11	107.8
Sn1—C2—H2A	109.5	C10—C11—H11	107.8
Sn1—C2—H2B	109.5	C11^i^—C11—H11	107.8
H2A—C2—H2B	109.5	C13—C12—C16	116.3 (3)
Sn1—C2—H2C	109.5	C13—C12—C11	121.8 (2)
H2A—C2—H2C	109.5	C16—C12—C11	121.9 (3)
H2B—C2—H2C	109.5	C12—C13—C14	120.1 (3)
Sn1—C3—H3A	109.5	C12—C13—H13	119.9
Sn1—C3—H3B	109.5	C14—C13—H13	119.9
H3A—C3—H3B	109.5	N3—C14—C13	124.2 (3)
Sn1—C3—H3C	109.5	N3—C14—H14	117.9
H3A—C3—H3C	109.5	C13—C14—H14	117.9
H3B—C3—H3C	109.5	N3—C15—C16	125.3 (3)
N1—C4—S1	178.0 (3)	N3—C15—H15	117.4
N2—C5—C6	123.3 (2)	C16—C15—H15	117.4
N2—C5—H5	118.3	C15—C16—C12	119.0 (3)
C6—C5—H5	118.3	C15—C16—H16	120.5
C5—C6—C7	119.8 (3)	C12—C16—H16	120.5
			
C1—Sn1—N1—C4	64.3 (14)	C7—C8—C9—N2	1.0 (5)
C3—Sn1—N1—C4	−175.1 (14)	C8—C7—C10—C11	−101.4 (3)
C2—Sn1—N1—C4	−54.0 (14)	C6—C7—C10—C11	79.9 (3)
C1—Sn1—N2—C5	24.7 (2)	C7—C10—C11—C12	62.2 (3)
C3—Sn1—N2—C5	−95.9 (2)	C7—C10—C11—C11^i^	−173.6 (3)
C2—Sn1—N2—C5	143.1 (2)	C10—C11—C12—C13	−123.3 (3)
C1—Sn1—N2—C9	−149.7 (3)	C11^i^—C11—C12—C13	112.7 (3)
C3—Sn1—N2—C9	89.7 (2)	C10—C11—C12—C16	57.5 (3)
C2—Sn1—N2—C9	−31.3 (2)	C11^i^—C11—C12—C16	−66.6 (4)
C9—N2—C5—C6	1.3 (4)	C16—C12—C13—C14	−0.3 (4)
Sn1—N2—C5—C6	−173.4 (2)	C11—C12—C13—C14	−179.6 (2)
N2—C5—C6—C7	0.1 (4)	C15—N3—C14—C13	−0.6 (5)
C5—C6—C7—C8	−1.0 (4)	C12—C13—C14—N3	0.5 (4)
C5—C6—C7—C10	177.9 (3)	C14—N3—C15—C16	0.5 (5)
C6—C7—C8—C9	0.4 (4)	N3—C15—C16—C12	−0.3 (6)
C10—C7—C8—C9	−178.4 (3)	C13—C12—C16—C15	0.2 (4)
C5—N2—C9—C8	−1.8 (4)	C11—C12—C16—C15	179.5 (3)
Sn1—N2—C9—C8	172.9 (2)		

Symmetry code: (i) −*x*, −*y*, −*z*.

### Hydrogen-bond geometry (Å, º)

Cg is the centroid of the N2-pyridine ring.

**Table:**

*D*—H···*A*	*D*—H	H···*A*	*D*···*A*	*D*—H···*A*
C14—H14···*Cg*^ii^	0.93	2.79	3.631 (4)	151

Symmetry code: (ii) −*x*+1, −*y*, −*z*.
